# Identification of immune-related biomarkers co-occurring in acute ischemic stroke and acute myocardial infarction

**DOI:** 10.3389/fneur.2023.1207795

**Published:** 2023-08-17

**Authors:** Shan Wang, Shengjun Tan, Fangni Chen, Yihua An

**Affiliations:** ^1^Emergency Station, Dougezhuang Community Health Service Center, Beijing, China; ^2^Key Laboratory of Zoological Systematics and Evolution, Institute of Zoology, Chinese Academy of Sciences, Beijing, China; ^3^Department of Nuclear Medicine, The Fifth Medical Center of the General Hospital of the People's Liberation Army, Beijing, China; ^4^Department of Neurosurgery, Sanbo Brain Hospital, Capital Medical University, Beijing, China

**Keywords:** acute ischemic stroke, acute myocardial infarction, immune response, neutrophils, CIBERSORT, weighted gene co-expression network analysis, bioinformatics

## Abstract

**Background:**

Acute ischemic stroke (AIS) and acute myocardial infarction (AMI) share several features on multiple levels. These two events may occur in conjunction or in rapid succession, and the occurrence of one event may increase the risk of the other. Owing to their similar pathophysiologies, we aimed to identify immune-related biomarkers common to AIS and AMI as potential therapeutic targets.

**Methods:**

We identified differentially expressed genes (DEGs) between the AIS and control groups, as well as AMI and control groups using microarray data (GSE16561 and GSE123342). A weighted gene co-expression network analysis (WGCNA) approach was used to identify hub genes associated with AIS and/or AMI progression. The intersection of the four gene sets identified key genes, which were subjected to functional enrichment and protein–protein interaction (PPI) network analyses. We confirmed the expression levels of hub genes using two sets of gene expression profiles (GSE58294 and GSE66360), and the ability of the genes to distinguish patients with AIS and/or AMI from control patients was assessed by calculating the receiver operating characteristic values. Finally, the investigation of transcription factor (TF)-, miRNA-, and drug–gene interactions led to the discovery of therapeutic candidates.

**Results:**

We identified 477 and 440 DEGs between the AIS and control groups and between the AMI and control groups, respectively. Using WGCNA, 2,776 and 2,811 genes in the key modules were identified for AIS and AMI, respectively. Sixty key genes were obtained from the intersection of the four gene sets, which were used to identify the 10 hub genes with the highest connection scores through PPI network analysis. Functional enrichment analysis revealed that the key genes were primarily involved in immunity-related processes. Finally, the upregulation of five hub genes was confirmed using two other datasets, and immune infiltration analysis revealed their correlation with certain immune cells. Regulatory network analyses indicated that *GATA2* and hsa-mir-27a-3p might be important regulators of these genes.

**Conclusion:**

Using comprehensive bioinformatics analyses, we identified five immune-related biomarkers that significantly contributed to the pathophysiological mechanisms of both AIS and AMI. These biomarkers can be used to monitor and prevent AIS after AMI, or vice versa.

## Introduction

Cardiovascular diseases (CVDs)—including stroke and ischemic heart diseases—pose a significant global health burden, affecting millions of people and causing substantial morbidity and mortality (1). Two of the most severe CVDs, acute ischemic stroke (AIS) and acute myocardial infarction (AMI), frequently become a heavy burden on families and society (2). Although the causes of AIS and AMI are unclear, their pathophysiologies are similar in principle: deficient blood and oxygen supply to the brain or heart. They are typically caused by sudden arterial blockage, which can be caused by the formation of a blood clot (thrombus) or plaque buildup (atherosclerosis). This blockage results in the deprivation of oxygen and nutrients to the surrounding tissues, leading to ischemia and necrosis of the affected tissues (3). The concurrence of AIS and AMI has also been reported in one patient (4, 5). They can occur simultaneously or in close temporal succession and are risk factors for one another (6). For example, the incidence of ischemic stroke (IS) after AMI is 4–5% (7, 8), while patients with AMI who concomitantly experience AIS are at a substantially higher risk of both in-hospital (>8-fold increase) and 1-year mortality (>3-fold increase) than patients with AMI alone (9). Similar treatments, such as reperfusion therapy or catheter-based thrombectomy, are used to treat AIS and AMI; however, these diseases occur suddenly and have a narrow therapeutic window. To improve patient outcomes, attempts, e.g., faster and more convenient diagnoses, are needed to shorten the treatment delay.

Inflammation is a key contributor to the development and progression of both cardiac and brain ischemia, and immune cells play a crucial role in the pathophysiology of CVDs as they are involved in inflammation and tissue injury (10–13). The systematic inflammatory response is activated after AIS or AMI and is involved in the entire process of these two diseases (14, 15). The neuroinflammatory response disrupts the blood–brain barrier in AIS, leading to the migration of macrophages, monocytes, lymphocytes, and other inflammatory cells to the ischemic site (16, 17). Studies have also shown that peripheral immune cells can contribute to secondary neurodegeneration after AIS by infiltrating the brain and interacting with resident brain cells (18). For AMI, various immune cells and genes participate in immunomodulation after an acute event, working together to rebuild injured areas and remove necrotic tissue (15). Chronic inflammation can also contribute to the development of atherosclerosis, which is a major risk factor for both conditions. Therefore, exploring the immune microenvironment and inflammatory mechanisms of AIS and AMI may identify potential immunoregulatory therapies as alternative treatment methods.

Genetic factors can influence the expression and activity of various immune and inflammatory molecules, which in turn can affect the severity and outcome of AIS and AMI. Certain genetic variants have been associated with an increased risk of AIS (19). Several studies have shown that dysregulated genes, long non-coding RNAs, and miRNAs are potential biomarkers of either AIS or AMI (20–22). For example, elevated expression of *MMP9* has been detected in patients with AMI when compared with controls, and plasma levels of *MMP9* and *NT-proBNP* have a time-dependent relationship (23). Understanding the genetic basis of immunoinflammatory mechanisms involved in AIS and AMI may help identify new therapeutic targets and improve patient outcomes. However, only a limited number of studies have focused on identifying biomarkers for the diagnosis of these diseases (14, 24). According to a family study, AIS and AMI share several genetic characteristics (25); therefore, there is an urgent need to screen for immune-related biomarkers of both diseases.

In our study, we acquired two datasets (GSE16561 and GSE123342) for identifying differentially expressed genes (DEGs) between individuals diagnosed with AIS or AMI and their respective control groups. Using weighted gene co-expression network analysis (WGCNA), we aimed to identify hub genes associated with AIS/AMI progression. Important genes were further analyzed using gene ontology (GO) and protein–protein interaction (PPI) network analyses, and CIBERSORT was used to analyze immune cell infiltration in AIS and AMI. Finally, the investigation of transcription factor (TF)-, miRNA-, and drug–gene interactions discovered the possible therapeutic candidates.

## Materials and methods

### Acquisition of expression data

**Figure 1:**
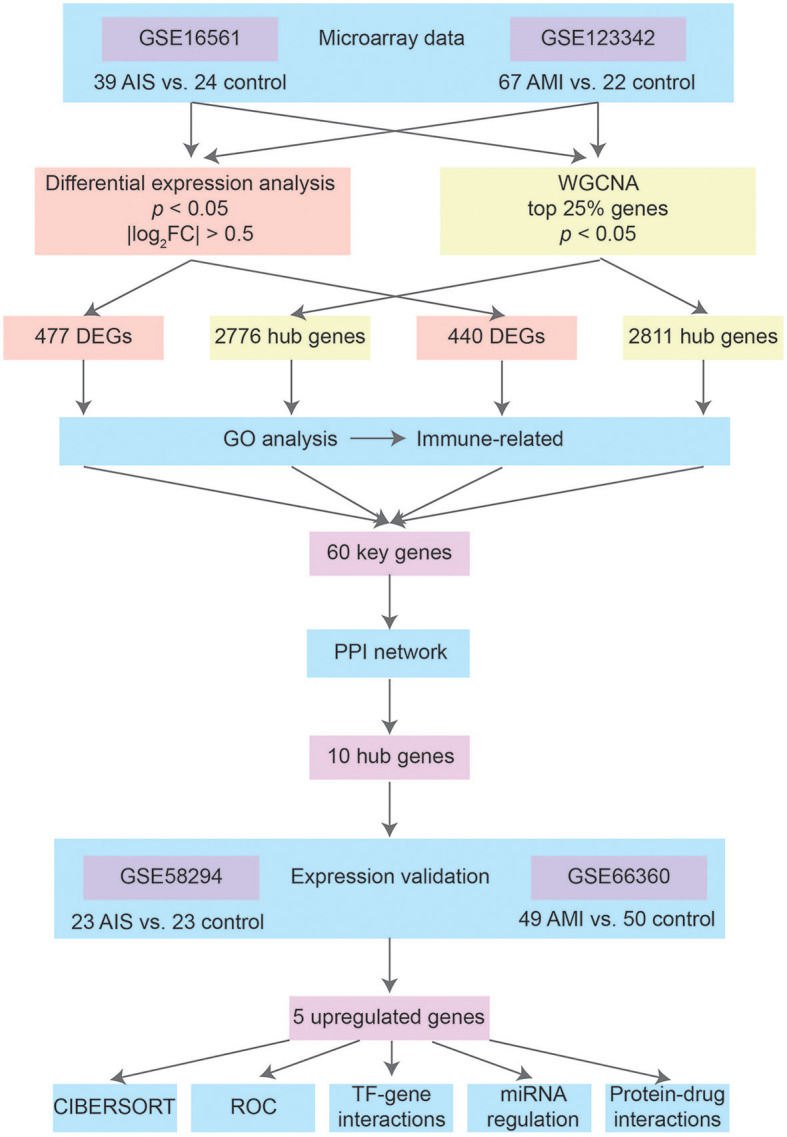
Workflow of the study.

Figure 1 shows the basic workflow of our study for identifying potential biomarkers of AIS and/or AMI. By searching for expression data related to AIS and AMI in the Gene Expression Omnibus (GEO) database (http://www.ncbi.nlm.nih.gov/geo/), we decided to focus on two datasets that contain sufficient samples to perform a comparative study: GSE16561 was acquired using the GPL6883 platform and contained a total of 39 AIS samples and 24 control samples; GSE123342 was obtained using the GPL17586 platform and consisted of 67 AMI samples and 22 control samples with stable coronary heart disease. These two datasets were used to identify key biomarkers of AIS and AMI.

We downloaded another two datasets from GEO to validate gene expression. GSE58294, which included 69 IS and 23 control samples, was created using the GPL570 platform. Onset among the 69 IS samples included three time points (3 h, 5 h, and 24 h). In this study, the 23 IS samples in the 3 h group were treated as AIS, while the 5 h and 24 h groups were treated as post-AIS. GSE66360, which included 49 patients with AMI and 50 healthy controls, was also created using the GPL570 platform.

GSE123342 contained additional myocardial infarction (MI) samples 30 days (*n* = 64) and 365 days (*n* = 37) after AMI. We used this dataset in conjunction with GSE66360 to investigate the temporal expression patterns of key genes identified in AIS and/or AMI.

### Data pre-processing and screening of DEGs

The microarray data were pre-processed before analysis. We found that the series matrix file of GSE16561 contained numerous NA values; therefore, we downloaded the raw profiling file. Expression values were then log2 transformed. For genes targeted by more than one probe, the median expression levels were calculated. We only retained protein-coding genes with a stable gene symbol and the Ensembl gene id; other genes, such as long non-coding RNAs and pseudogenes, were excluded.

The identification of DEGs in GSE16561 and GSE123342 was based on the limma package in R. DEGs in AIS and AMI were filtered using the following cutoff criteria: an adjusted *p*-value of < 0.05 and |log_2_FC| > 0.5.

### Construction of WGCNA

The gene expression matrix was standardized by scaling after pre-processing. Subsequently, the WGCNA package in R was used to identify hub genes. The initial dataset consisted of the highest variance genes, which comprised the top 25% of genes in the normalized gene expression matrix file. The samples were clustered using the average linkage method in WGCNA. The scale independence and average connectivity were calculated and used to obtain a scale-free network. The similarity matrix was converted into an adjacency matrix, which was then used to calculate the topological overlap matrix (TOM) values. Genes were hierarchically clustered based on the dissimilarity measure (1-TOM) derived from the TOM values, and the dynamic tree-cut (DTC) method was used to identify modules. The minimum module size for the resulting dendrogram was set to 30 genes. Close modules with a threshold of 0.25 were merged.

### Functional enrichment analysis

After retrieving four gene sets, the DEGs of AIS and AMI and hub genes of AIS and AMI in the key modules of WGCNA, the clusterProfiler R package was used to perform functional enrichment analysis, i.e., GO. Significantly enriched terms were identified based on an adjusted *p*-value of < 0.01. GO enrichment analysis included biological processes (BPs), cellular components (CCs), and molecular functions (MFs). Common GO terms among the four gene sets were identified by overlapping the aforementioned results.

### PPI network

From the four gene sets, 60 key genes were identified and subjected to the construction of the PPI network in the STRING database (26), where a threshold of 0.4 was set as the minimum confidence interaction score. The PPI network was visualized and analyzed using Cytoscape 3.9.1 (27). Functional enrichment analyses of the PPI network, including BP analysis, Reactome pathway analysis, and annotated keywords in UniProt were conducted using STRING. The MCC method in Cytoscape was employed to identify the 10 hub genes with the highest connection scores.

### Immune cell infiltration analysis

The CIBERSORT algorithm is a widely used computational tool that enables the estimation of the infiltration levels of 22 different immune cell types in various diseases. We downloaded the default LM22 signature matrix file and R package according to the instructions on the CIBERSORT website. The relative proportion of immune cells was calculated for AIS and AMI samples, and Spearman's correlation coefficient was used to determine the strength and direction of the relationship between genes and immune cells.

### TF-, miRNA-, and drug–gene interaction analyses

After validation, five upregulated genes in AIS and AMI were selected as target genes. Three networks—including the TF-gene, miRNA-gene, and drug–protein interaction networks—were analyzed for the target genes in NetworkAnalyst using the JASPAR, TarBase (version 8.0), and DrugBank (version 5.0) software packages.

## Results

### DEGs in AIS and AMI

**Figure 2:**
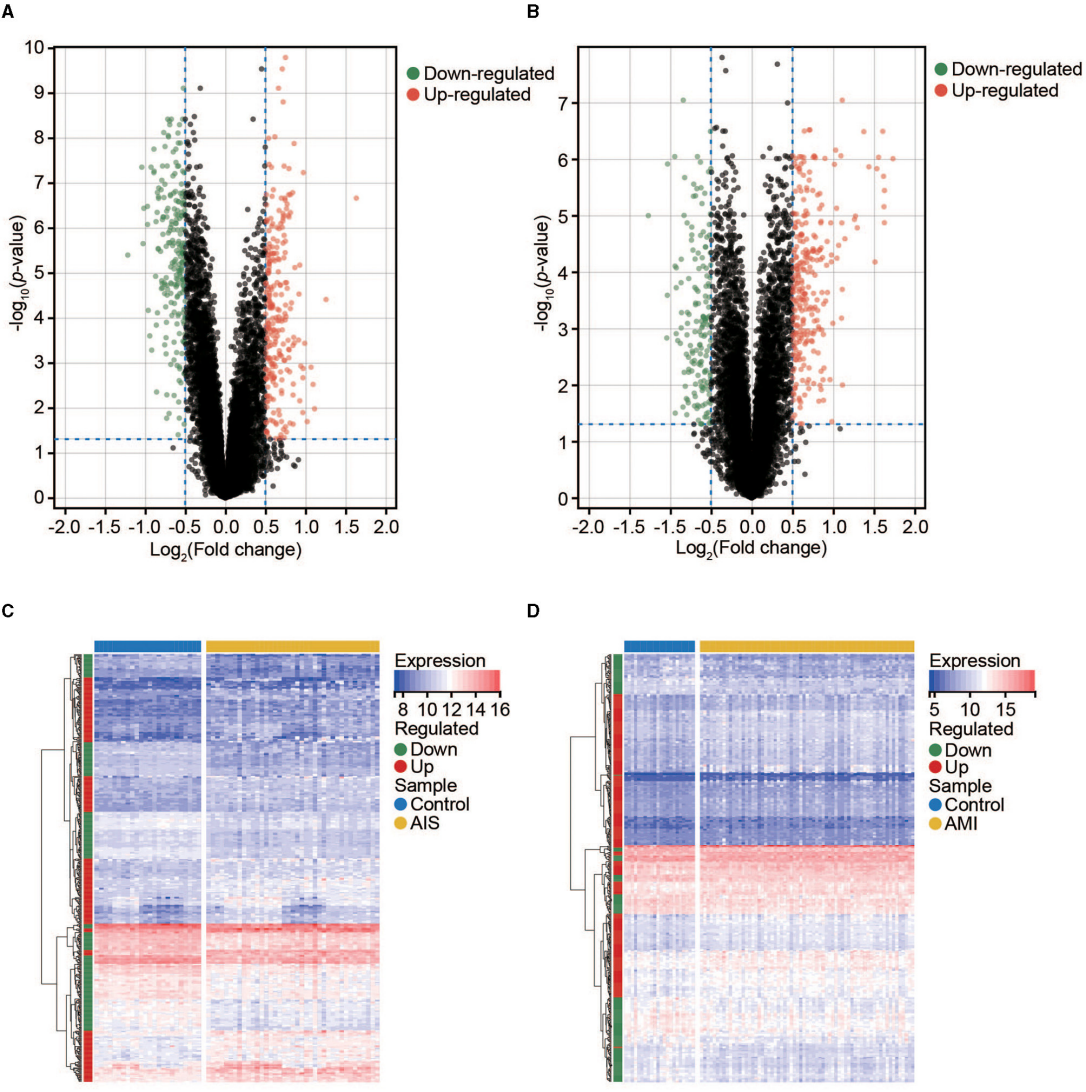
Expression profile of DEGs in AIS and AMI. **(A)** Volcano map of DEGs in AIS. **(B)** Volcano map of DEGs in AMI. **(C)** Clustered heatmap of DEGs in AIS. **(D)** Clustered heatmap of DEGs in AMI.

We used the limma package in R to conduct DEG analysis on the microarray transcriptome data of the AIS and control samples or AMI and control samples. In total, 477 and 440 DEGs were identified in the AIS and AMI samples, respectively (Figures 2A, B). The expression profiles of these DEGs are shown in Figures 2C, D; among them, 225 were downregulated and 252 were upregulated in the AIS samples, whereas 165 were downregulated and 275 were upregulated in the AMI samples (Figures 2A, B). These DEGs were further considered to be candidate transcriptional signatures.

### Key modules and hub genes

**Figure 3:**
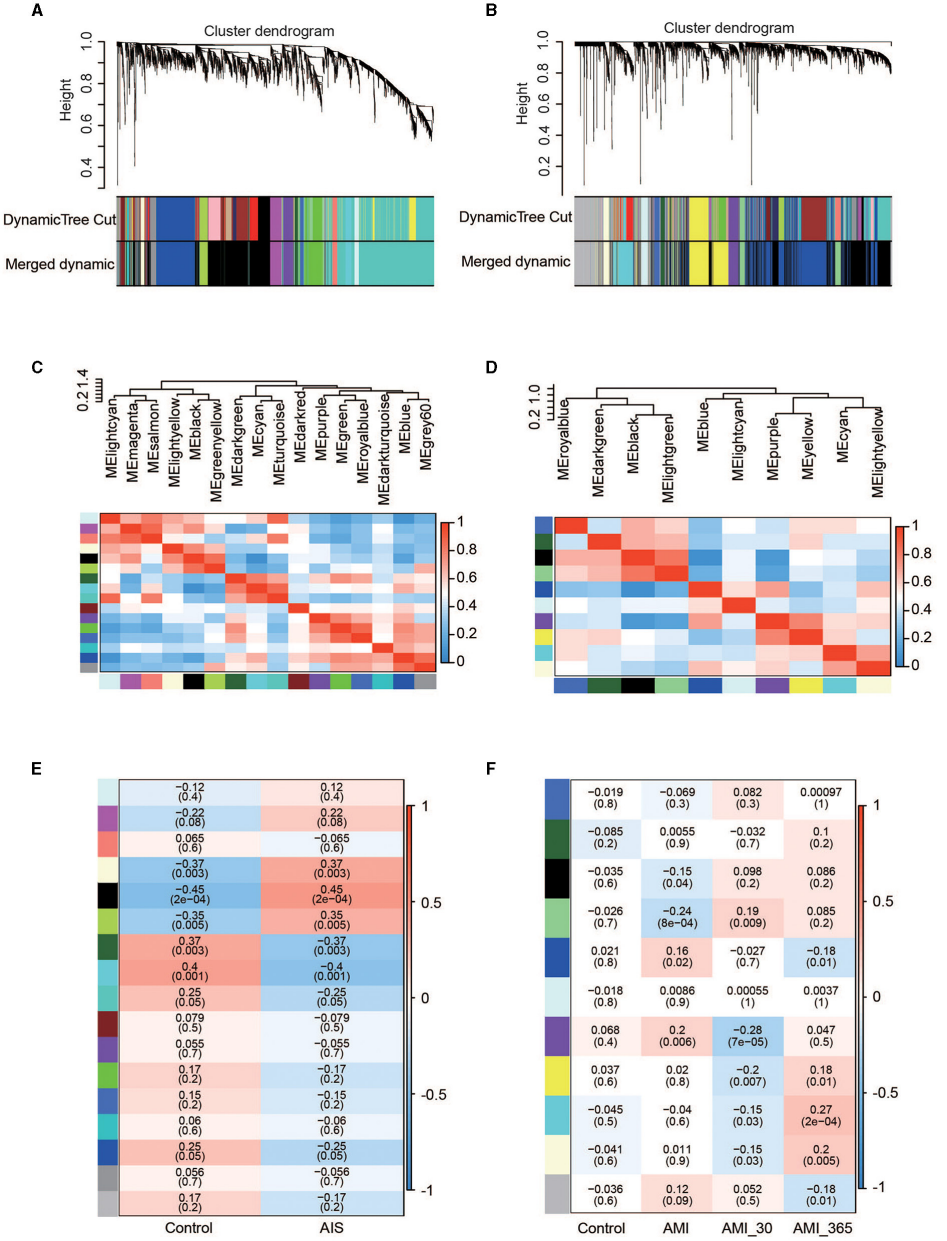
Co-expression networks in AIS and AMI. **(A)** Gene dendrogram obtained by average linkage hierarchical clustering in AIS. The row underneath the dendrogram shows the module assignment determined by the dynamic tree cut. **(B)** Gene dendrogram obtained by average linkage hierarchical clustering in AMI. **(C)** Relationship among all modules in AIS. **(D)** Relationship among all modules in AMI. **(E)** Correlation coefficients of the WGCNA modules between the control and AIS groups. **(F)** Correlation coefficients of the WGCNA modules between the control, AMI, and post-AMI groups.

To identify groups of genes with highly correlated expression patterns across the AIS and AMI samples, we performed a co-expression analysis of all genes using the WGCNA R package. As no obvious outlier samples were detected in the sample clustering, we did not exclude any samples from the subsequent WGCNA. The top 25% of genes with the highest degree of variation in both datasets were subsequently chosen as the input. We selected soft thresholds of 7 for AIS and 10 for AMI when *R*^2^ > 0.85 (Supplementary Figure 1) and identified 16 and 10 modules, respectively (Figures 3A–D). Based on the correlation coefficients between the sample groups and modules, we selected the key modules as those significantly related to AIS and AMI (Figures 3E, F). A total of 2,776 and 2,811 genes were incorporated into these key modules, respectively.

### Functional enrichment analysis

**Figure 4:**
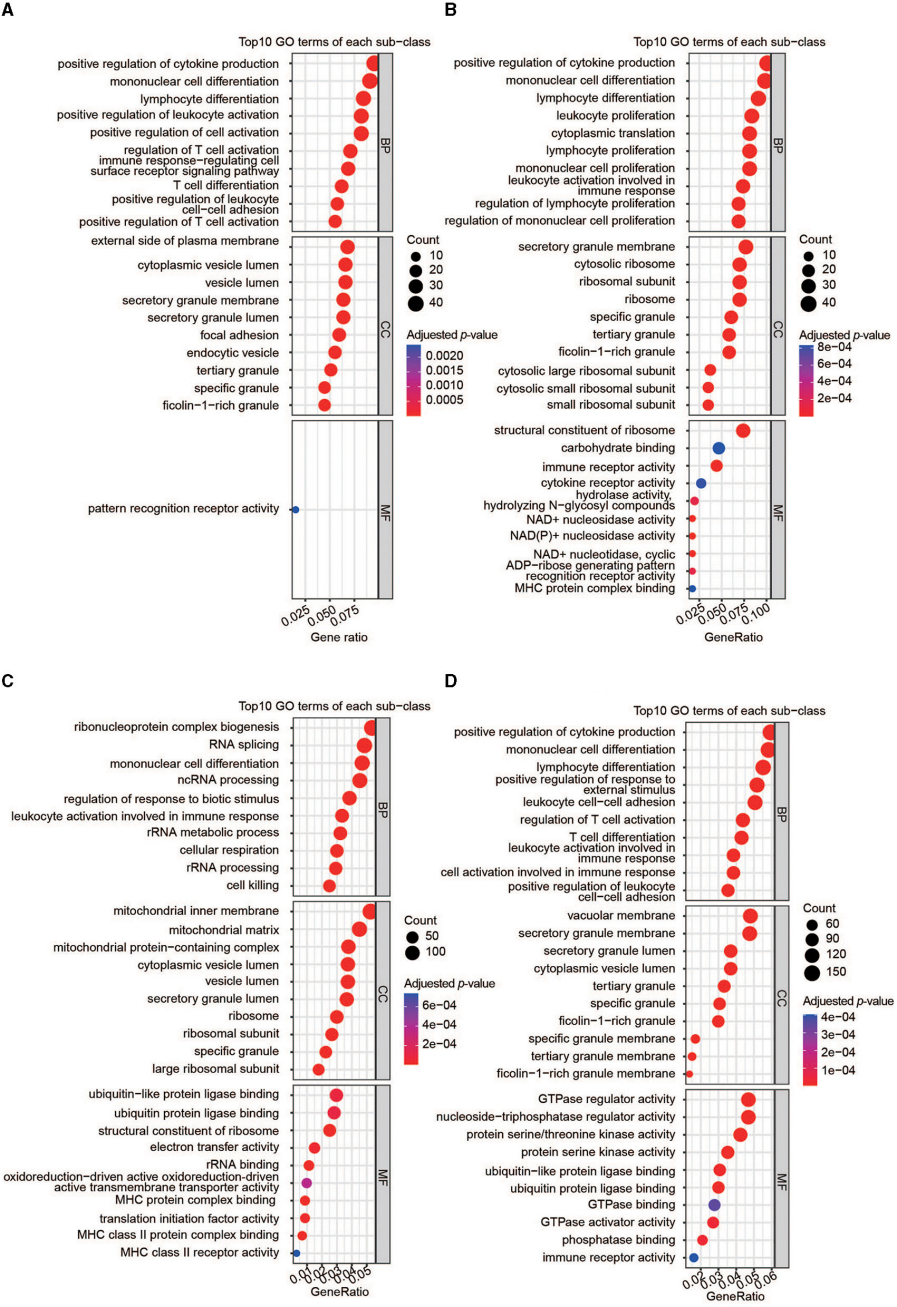
Functional analyses of the important genes. **(A)** GO enrichment analysis of DEGs in AIS. **(B)** GO enrichment analysis of DEGs in AMI. **(C)** GO enrichment analysis of hub genes in AIS. **(D)** GO enrichment analysis of hub genes in AMI.

Four gene sets were included for gene enrichment analysis: the DEGs and hub genes identified through WGCNA in AIS and AMI. Generally, the most important genes are enriched in immune-related processes (Figures 4A–D). We then selected the most common GO terms among the four gene lists, with a *p*-value of < 0.01 as the cutoff. Among the 77 BP terms shared by all four datasets, 54 (70.1%) were related to immunity (Supplementary Table 1), for example, positive regulation of cytokine production, lymphocyte differentiation, positive regulation of leukocyte activation, regulation of T-cell activation, and leukocyte-mediated immunity. CC outcomes showed that most proteins were located on the membrane (Supplementary Table 1), suggesting that they may participate in immune responses.

### PPI of the key genes

**Figure 5:**
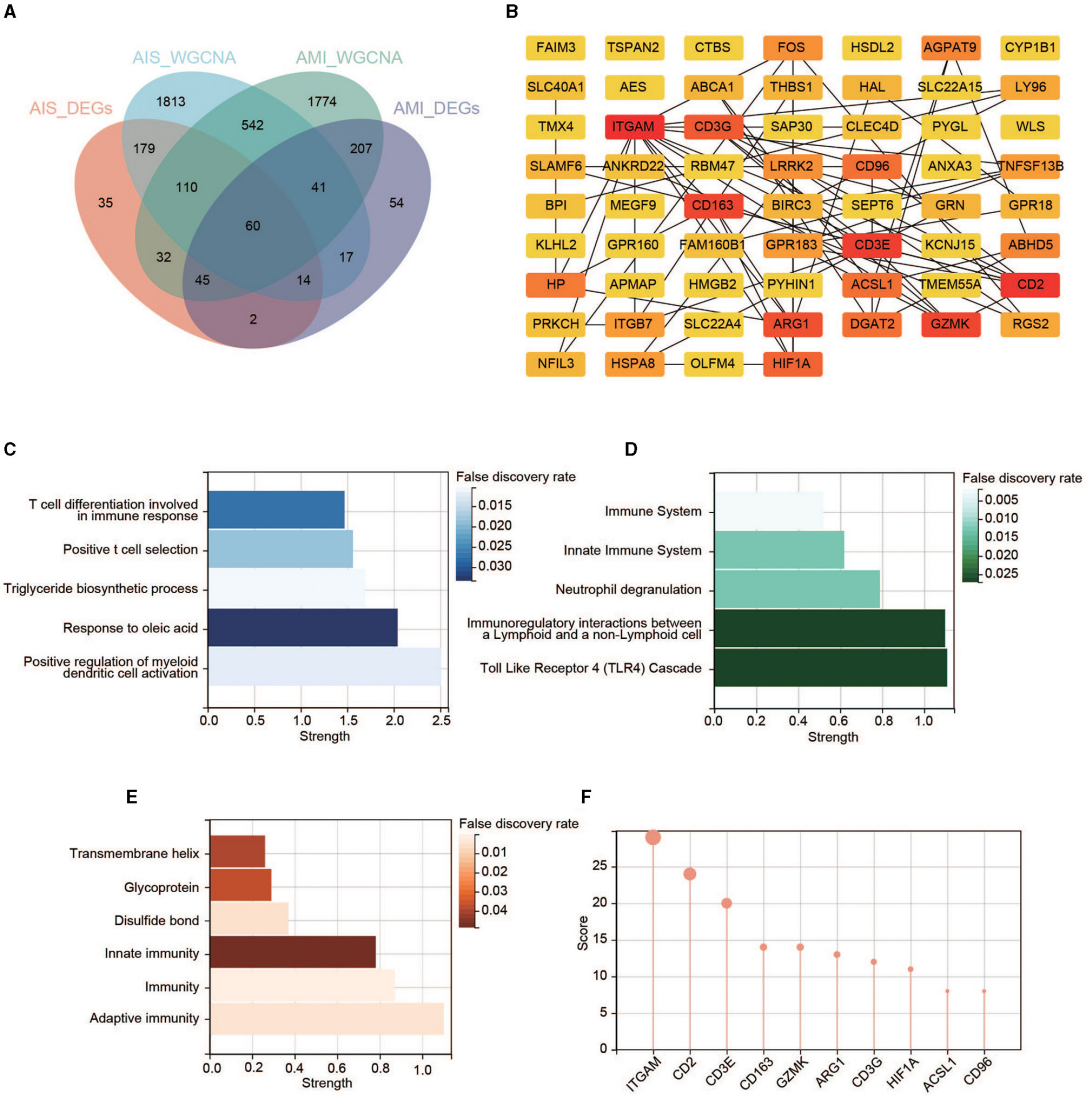
Identification of the key genes. **(A)** Venn diagram of the four gene sets. **(B)** PPI network of the 60 key genes. Colors from yellow to red represent the low-to-high MCC score. **(C)** GO enrichment analysis in the PPI network. **(D)** Reactome pathway analysis in the PPI network. **(E)** Annotated keywords in UniProt enrichment analysis in the PPI network. **(F)** Top 10 genes in the PPI network ranked by the MCC method.

Overlaps between the four datasets identified 60 key genes (Figure 5A), with 60 nodes and 63 edges in the PPI network (Figure 5B and Supplementary Figure 1). Functional enrichment in the network also demonstrated that the key genes were immune-related. The top five BP terms were T-cell differentiation involved in immune response, positive T-cell selection, triglyceride biosynthetic process, response to oleic acid, and positive regulation of myeloid dendritic cell activation (Figure 5C). The significantly enriched Reactome pathways included the immune system, innate immune system, neutrophil degranulation, immunoregulatory interactions between a lymphoid and a non-lymphoid cell, and toll-like receptor 4 (TLR4) cascade (Figure 5D). Significantly enriched annotated keywords in UniProt were transmembrane helix, glycoprotein, disulfide bond, innate immunity, immunity, and adaptive immunity (Figure 5E). Finally, a topological analysis helped identify the top 10 hub genes: *ITGAM, CD2, CD3E, CD163, GZMK, ARG1, CD3G, HIF1A, ACSL1*, and *CD96* (Figure 5F).

### Validation of the hub genes

**Figure 6:**
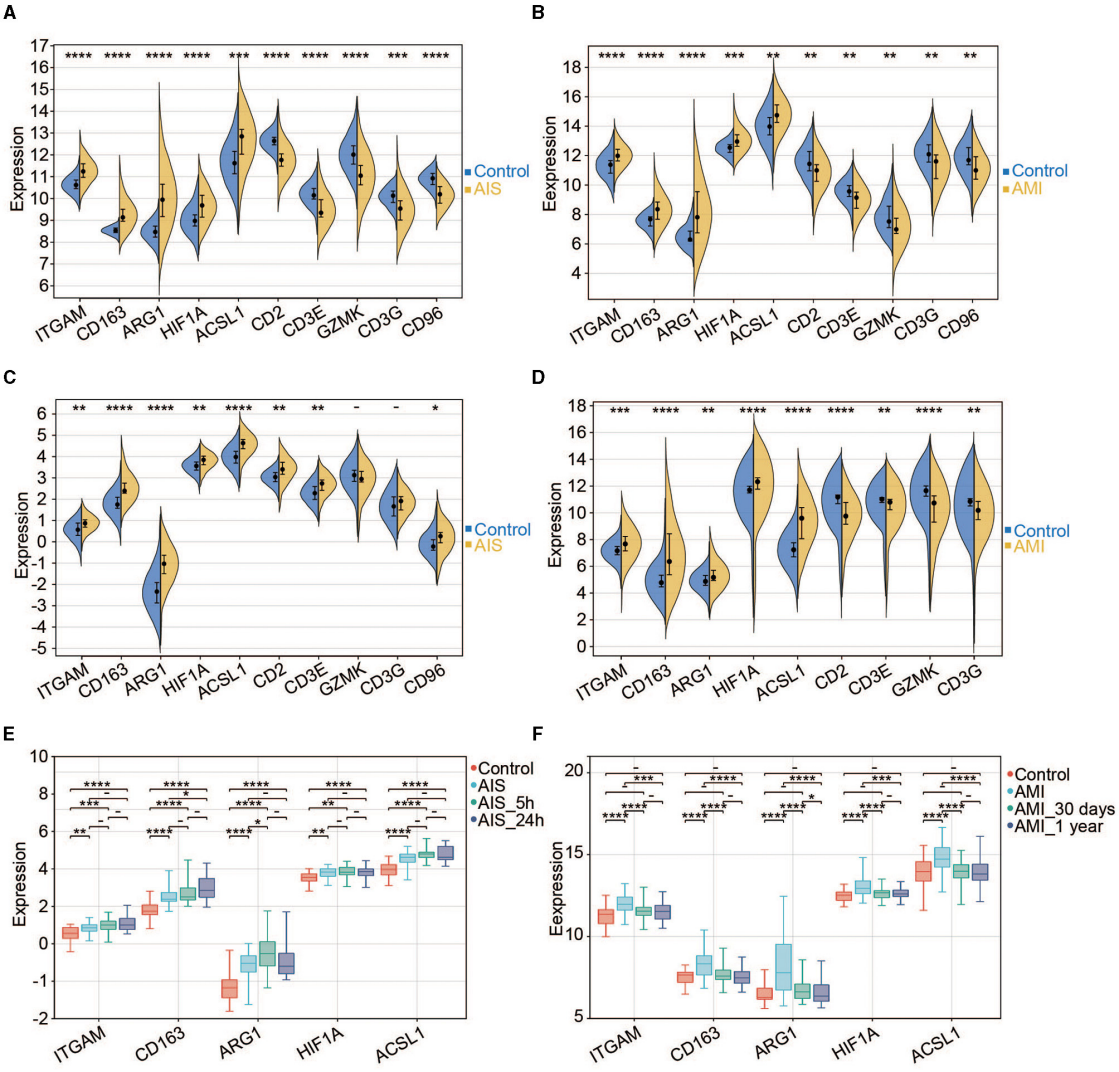
Expression pattern of the hub genes. **(A)** Expression profiles of the 10 hub genes in the control and AIS groups (GSE16561). **(B)** Expression profiles of the 10 hub genes in the control and AMI groups (GSE123342). **(C)** Expression profiles of the 10 hub genes in the control and AIS groups (GSE58294). **(D)** Expression profiles of the nine hub genes in the control and AIS groups (GSE66360). *CD96* does not have a probe in GSE66360. **(E)** Expression profiles of the five hub genes among different time points for AIS (GSE58294). **(F)** Expression profiles of the five hub genes among different time points for AMI (GSE123342). ^*^P < 0.05, ^**^P < 0.01, ^***^P < 0.001, and ^****^P < 0.0001.

We found that five hub genes were upregulated (expressed at a significantly higher level in patients with AIS/AMI than in control samples), whereas five other genes were significantly downregulated in patients with AIS/AMI (Figures 6A, B). The expression patterns of these genes were validated using two other microarray transcriptome datasets from patients with AIS and AMI. The upregulated expression patterns of *ITGAM, CD163, ARG1, HIF1A*, and *ACSL1* were confirmed in both datasets (Figures 6C, D). Moreover, samples from GSE58294 were classified into three time groups: 3 h, 5 h, and 24 h after AIS. The results showed that these genes were consistently upregulated in all three IS groups compared with the control group; the highest expression level was usually reached at 5 h post-AIS (Figure 6E). Similarly, samples from GSE123342 were classified into three time groups: acute phase, 30 days after AMI, and 365 days after AMI. Five genes were upregulated in the acute phase, and their expression levels decreased to normal after 30 days when compared with the control group (Figure 6F). Combining these results, we concluded that the high expression of these genes can last up to 24 h, after which their expression levels begin to decrease to normal levels.

### The potential of hub genes as diagnostic markers

**Figure 7:**
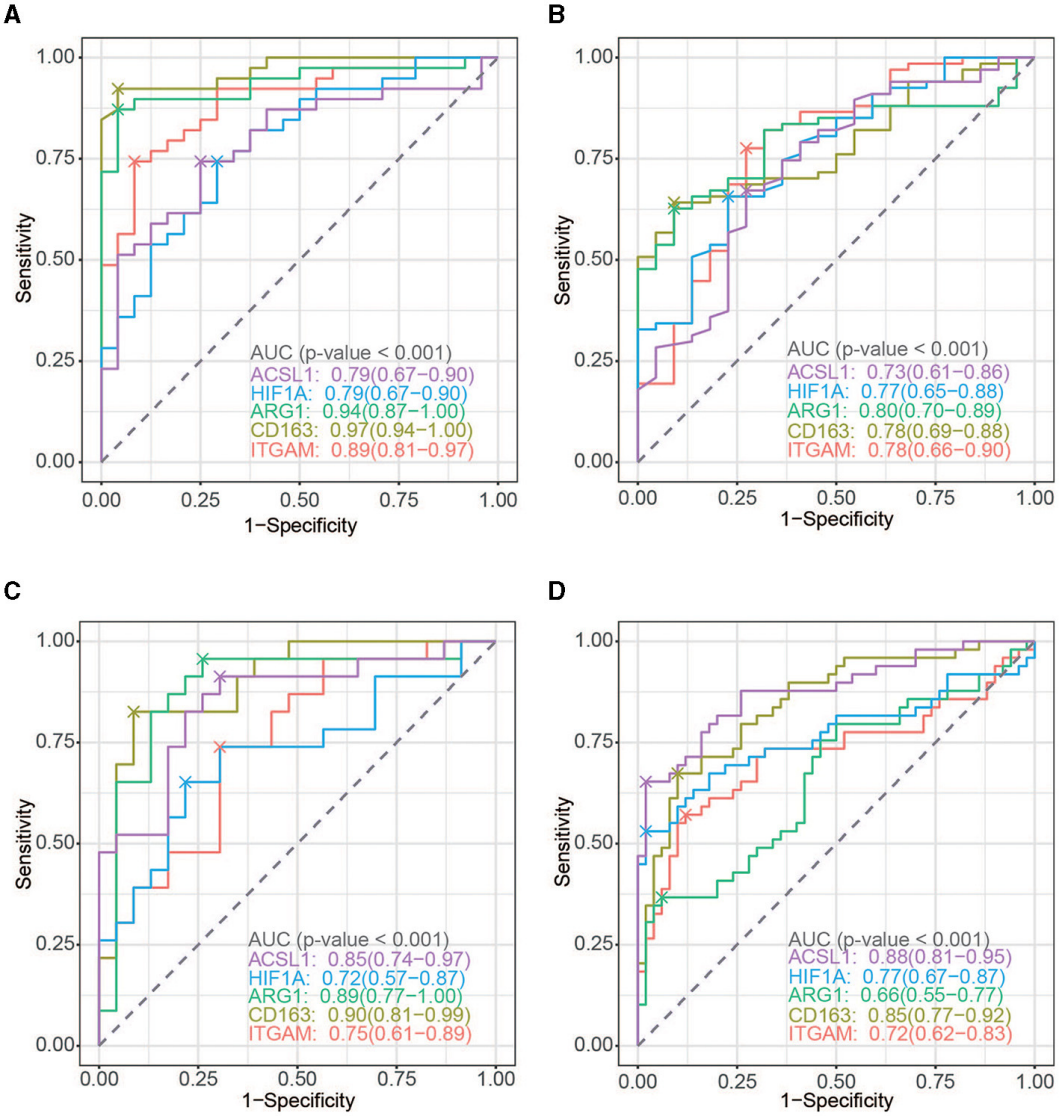
ROC curve analysis. **(A)** ROC curve of the five hub genes in AIS (GSE16561). **(B)** ROC curve of the five hub genes in AMI (GSE123342). **(C)** ROC curve of the five hub genes in AIS (GSE58294). **(D)** ROC curve of the five hub genes in AIS (GSE66360).

To evaluate the diagnostic power of the five immune-related biomarkers for AIS and AMI, receiver operating characteristic (ROC) analysis was performed on multiple datasets. The AUC values were then obtained for ITGAM, CD163, ARG1, HIF1A, and ACSL1, which were 0.89, 0.97, 0.94, 0.79, and 0.79 in GSE16561 (Figure 7A); 0.78, 0.78, 0.80, 0.77, and 0.73 in GSE58294 (Figure 7B); 0.75, 0.90, 0.89, 0.72, and 0.85 in GSE123342 (Figure 7C); and 0.72, 0.85, 0.66, 0.77, and 0.88 in GSE66360 (Figure 7D), respectively.

### Immune infiltration analysis

**Figure 8:**
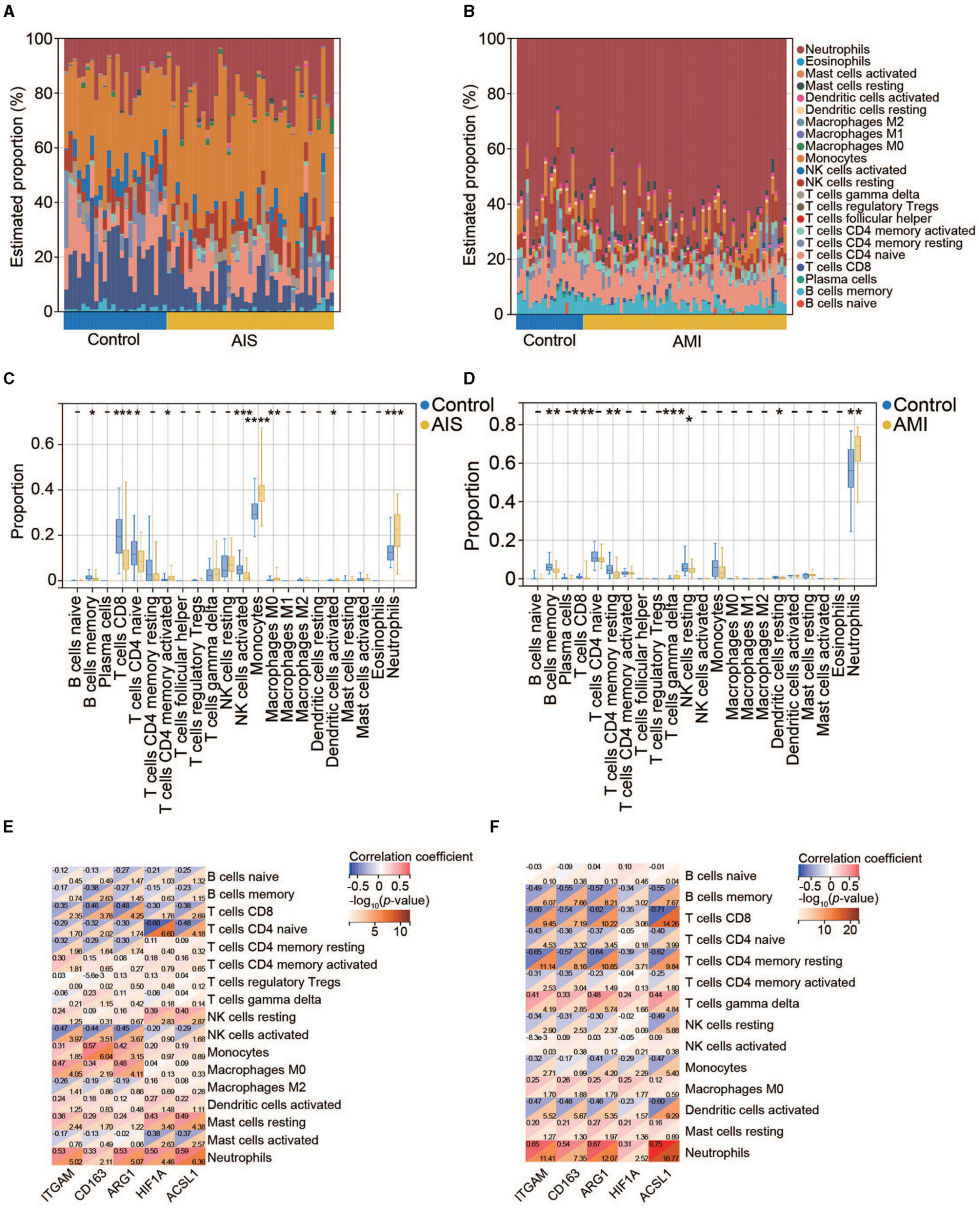
Immune cell infiltration analysis. **(A)** Percentage distribution of 22 immune cell subtypes in GSE16561. **(B)** Percentage distribution of 22 immune cell subtypes in GSE123342. **(C)** The difference in immune cell infiltration between the control and AIS groups. **(D)** The difference in immune cell infiltration between the control and AMI groups. **(E)** Correlation between the five hub genes and immune cells in AIS. **(F)** Correlation between the five hub genes and immune cells in AMI.

To further investigate the significance of the identified genes, we examined the levels of infiltrating immune cells in AIS and AMI as suggested by GO analysis, highlighting their immune-related functionality. In the AIS samples, the most prominent infiltrating immune cells were monocytes, neutrophils, and CD8+ T cells (Figure 8A); the AMI samples were characterized by an abundance of neutrophils (Figure 8B). Compared with the control group, patients with AIS and AMI exhibited significantly elevated levels of neutrophils and lower levels of memory B cells and CD8+ T cells (Figures 8C, D). Investigation of the relationship between the five genes and immune cells revealed that all genes exhibited a significantly positive correlation with neutrophils and a significantly negative correlation with CD8+ T cells in both the AIS and AMI samples (Figures 8E, F).

### Construction of TF-, miRNA-, and drug–gene interactions

**Figure 9:**
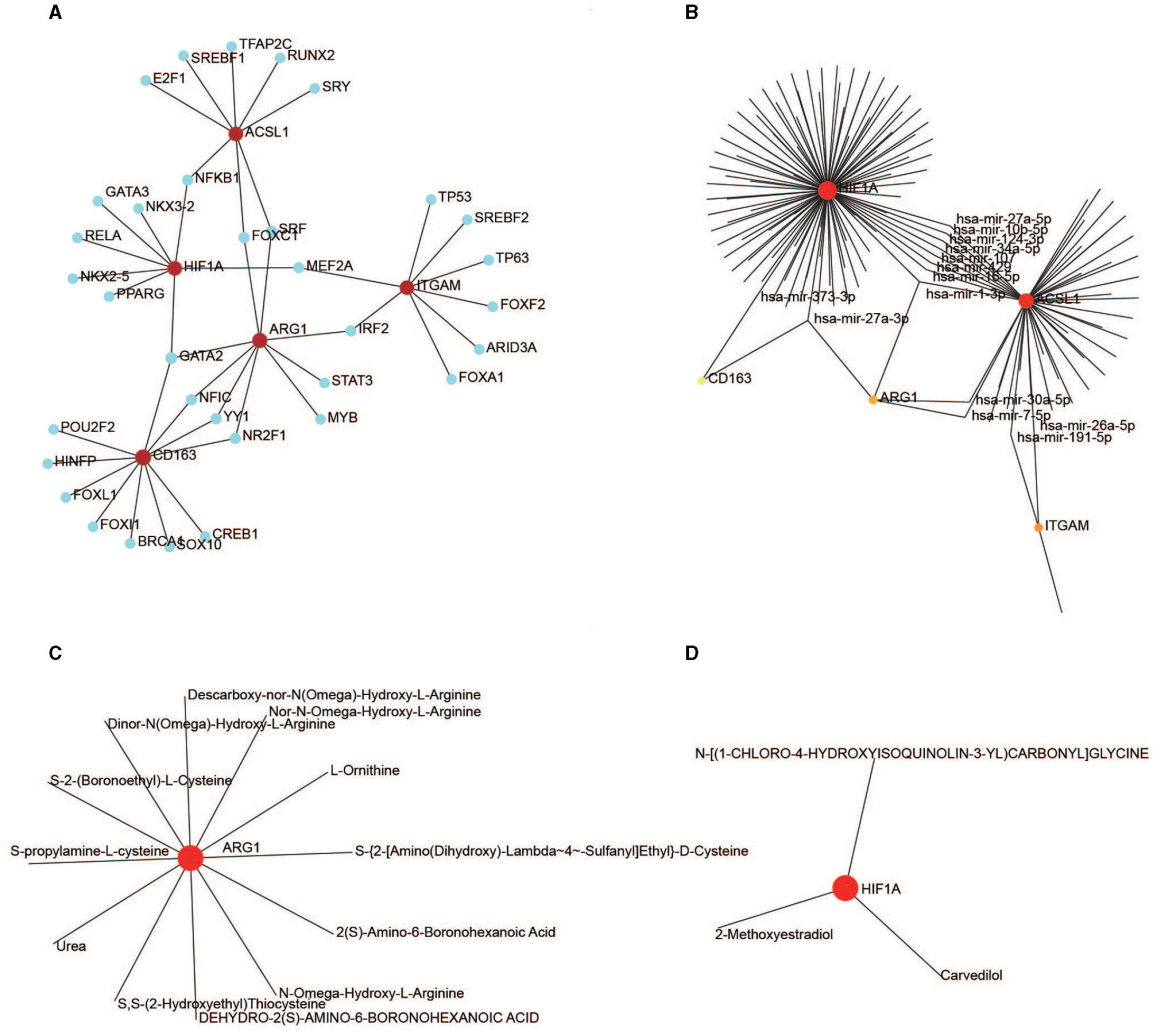
Regulation of the hub genes. **(A)** TF-gene interaction for the five hub genes. **(B)** MiRNA-gene interaction for the five hub genes. **(C)** Drug-ARG1 interaction. **(D)** Drug-HIF1A interaction.

We identified 34 TFs that targeted the five hub genes using the JASPAR software package in NetworkAnalyst. Nine key TFs—*GATA2, NR2F1, FOXC1, YY1, MEF2A, NFIC, SRF, NFKB1*, and *IRF2*—have a node degree value of ≥2 (Figure 9A).

One hundred and thirty-eight miRNAs targeting the five hub genes were obtained from NetworkAnalyst using TarBase. Hsa-mir-27a-3p and hsa-mir-1-3p targeted three genes, whereas hsa-mir-30a-5p, hsa-mir-107, hsa-mir-7-5p, hsa-mir-34a-5p, hsa-mir-191-5p, hsa-mir-429, hsa-mir-10b-5p, hsa-mir-373-3p, hsa-mir-124-3p, hsa-mir-16-5p, hsa-mir-27a-5p, and hsa-mir-26a-5p targeted two genes (Figure 9B).

Drug-targeting proteins encoded by *ARG1* and *HIF1A* were identified in NetworkAnalyst using DrugBank (version 5.0). Eleven therapeutic drugs interacted with ARG1 (Figure 9C), and three drugs interacted with HIF1A (Figure 9D); no drugs targeted ITGAM, CD163, or ACSL1.

## Discussion

As two of the most prominent causes of mortality and disability worldwide, AIS and AMI share several genetic characteristics (25). A growing consensus has been reached regarding the importance of early prevention of AIS and AMI. Microarray analysis is a valuable tool for identifying susceptibility genes for AIS and AMI and may ultimately lead to improved diagnosis, prevention, and treatment of the disease. In the present study, we identified five hub genes (*ITGAM, CD163, ARG1, HIF1A*, and *ACSL1*) using integrated analyses of AIS and AMI datasets, including DEG, WGCNA, GO enrichment, PPI network, and regulatory network analyses. We also verified the upregulation of these five genes in AIS and AMI samples. On the one hand, these identified genes have the potential to serve as biomarkers for the diagnosis of patients with AIS or AMI. On the other hand, studies have shown that these diseases could be risk factors for one another. Therefore, these biomarkers can be used to monitor and prevent AIS after AMI or vice versa.

Atherosclerosis, characterized by inflammatory cell accumulation in the arterial walls, is a well-known instance of chronic arterial inflammation and is commonly regarded as the pathological foundation for both AIS and AMI (28). The arterial narrowing can result in decreased blood flow and oxygen supply to the heart muscle, ultimately leading to the development of AIS and/or AMI. Immune cells play a fundamental role in the pathophysiology of atherosclerosis (10), and there is a genetic basis for the inflammatory pathogenesis of AIS and/or AMI. For example, the plasma levels of specific immune-inflammatory markers were reduced with atorvastatin treatment in AIS (29). Certain KIR genes and HLA alleles may modulate cytokine and cell-mediated inflammatory activation, which could contribute to stroke occurrence and severity after AIS (19, 30). Similarly, we found that important genes (DEG and WGCNA) were always enriched in immune responses, representing an important medium between inflammation and atherosclerosis. They are involved in the regulation of multiple immune cell types, such as B cells, T cells, lymphocytes, and leukocytes (Figure 4), suggesting that the migration of these cells to AIS and AMI sites may release pro-inflammatory factors and help disrupt the blood barrier.

When concentrating on the Reactome enrichment of the PPI network, several signaling pathways were detected, such as the TLR4 cascade and neutrophil degranulation (Figure 5D). Activation of TLR4 triggers the biosynthesis of diverse mediators of inflammation (31), and neutrophil degranulation is a common feature of many inflammatory disorders, including AIS and AMI (32). Coincidentally, we found a significantly greater proportion of neutrophils in the AIS/AMI group than in the control group (Figures 8C, D). Neutrophils are the first to be recruited to AIS and AMI sites (33, 34) and have pathophysiological relevance in AIS and AMI; for example, the presence of neutrophils in the brain can exacerbate impairment of the blood–brain barrier. We found that gene expression levels were significantly positively correlated with the proportion of neutrophils among the 22 immune cells (Figures 8E, F). For the first time, we demonstrated that immune modulation by neutrophils in AIS and AMI could potentially target ITGAM, CD163, ARG1, HIF1A, and ACSL1, thereby establishing a theoretical rationale for immune-targeted interventions in AIS and AMI.

The immune functions of these five genes in AIS and AMI were partially elucidated in previous studies. *ITGAM* encodes the integrin alpha M chain, which binds neutrophils and monocytes to the stimulated endothelium; additionally, it was demonstrated to function as a receptor for complement component 3, thereby contributing to the inflammatory response (35). In particular, the upregulation of *ITGAM* expression, which indicates increased inflammatory activation of immune cells, has been observed in patients with thrombus (36) and is thus associated with AIS and AMI (37, 38).

CD163, which is primarily expressed by monocytes and macrophages, serves as a scavenger of haptoglobin–hemoglobin complexes. Specifically, CD163 is considered a marker of alternatively activated or anti-inflammatory macrophages. Studies have shown that the soluble form of CD163 could be a potential biomarker in AIS and AMI (39, 40).

ARG1 is an enzyme that can modulate the synthesis of nitric oxide (NO) in the immune system. By suppressing the release of NO from macrophages, ARG1 can inhibit the production of pro-inflammatory cytokines (41). Similar bioinformatics-based approaches identified ARG1 as a potential biomarker in AIS and AMI (24, 42).

*HIF1A* is a TF that governs oxygen availability during inflammatory responses in the pathogenesis of AIS. Moreover, it is responsible for NLRP3 inflammasome-initiated pyroptosis following IS (43). An experiment in transgenic mice demonstrated that overexpression of *HIF1A* led to reduced infarct size and enhanced cardiac function 4 weeks after AMI (44).

Elevated triglyceride levels were observed in the peripheral white blood cells of patients with AMI. This finding can be attributed to the upregulation of *ACSL1*, which suppresses fatty acid β-oxidation via the PPARγ pathway, resulting in increased triglyceride levels (45). However, the functional role of *ACSL1* in AIS remains still unclear.

Our investigation revealed that gene expression levels increased within the first 3–24 h of AIS onset (Figure 6E). Consistently, another study based on microarray data showed that a comprehensive alteration in the gene expression profile, including that of *ARG1*, was discernible in the peripheral blood cells of patients with AIS within 3–24 h after onset (46). Subsequently, the gene expression decreased to normal levels after 30 days (Figure 6F), indicating an instantaneous role of these genes after AMI. In the future, sequencing data of both AIS and AMI at more time points should be obtained to reveal clearer molecular dynamics and physiological details during IS and MI development.

In our study, we found that GATA2 interacts with three biomarkers: ARG1, CD163, and HIF1A. Similarly, hsa-mir-27a-3p regulates these three biomarkers. As a vital TF in multilineage hematopoiesis, mutations in *GATA2* induce several hematological diseases (47). *GATA2* is upregulated in ischemia-reperfusion injury (48); additionally, there is a link between GATA2 deficiency and AIS (49). Interestingly, hsa-mir-27a-3p alleviates cerebral ischemia-reperfusion injury by targeting FOXO1 (50), therefore playing a significant therapeutic role in the management of AIS. According to our results, both GATA2 and hsa-mir-27a-3p can target ARG1, CD163, and HIF1A; thus, it is possible that they may function in the same pathway in AIS and AMI. Further investigations are required to elucidate the mechanism by which GATA2 and hsa-mir-27a-3p co-modulate ARG1, CD163, and HIF1A expression in AIS and AMI.

We identified 12 drugs targeting HIF1A, and three drugs targeting ARG1, which have therapeutic potential to treat patients with AIS and AMI. Conducting a range of laboratory-based trials can thus facilitate the determination of the efficacy of a compound and offer alternative solutions to immunotherapy for AIS and AMI.

In conclusion, we have addressed the scarcity of studies investigating common biomarkers derived from the shared pathological characteristics of both AIS and AMI using comprehensive bioinformatics analyses. Second, we have delved into potential targets for five biomarkers in the immune microenvironment of AIS and AMI, such as neutrophils, which expanded our understanding of these diseases. Third, while previous studies have provided partial elucidation of these five biomarkers primarily through experiments, we not only confirmed their importance in the pathophysiology of AIS and AMI but also established the value of microarray analysis for identifying susceptibility genes associated with both conditions. Finally, the identification of GATA2 and hsa-mir-27a-3p as agents capable of targeting ARG1, CD163, and HIF1A suggest that these two elements may function within the same pathway in both AIS and AMI.

There are limitations in our study as well. The analysis was conducted using data from public databases that originated from various platforms, which had different inclusion criteria and lacked corresponding clinical data in general. Additionally, it is important to note that our study is confined to the transcriptome level, and further validation of the findings is necessary through prospective clinical and basic experiments.

## Data availability statement

The original contributions presented in the study are included in the article/Supplementary material, further inquiries can be directed to the corresponding author.

## Ethics statement

Ethical review and approval was not required for the study on human participants in accordance with the local legislation and institutional requirements. Written informed consent from the patients/participants or patients/participants' legal guardian/next of kin was not required to participate in this study in accordance with the national legislation and the institutional requirements.

## Author contributions

YA and SW designed the study and wrote the manuscript. ST performed the data analysis. FC revised the manuscript. All authors contributed to the article and approved the submitted version.
