# Supplementary material for: Identification of immune-related biomarkers co-occurring in acute ischemic stroke and acute myocardial infarction
Source: Front Neurol. 2023 Aug 17;14:1207795. doi: 10.3389/fneur.2023.1207795 (PMC10469875; doi:10.3389/fneur.2023.1207795)
Supplement: Supplementary file 1 [file Data_Sheet_1.PDF]

Supplementary Material

Identification of immune-related biomarkers co-occurring in acute ischaemic stroke and acute myocardial infarction

Shan Wang[1], Shengjun Tan[2], Fangni Chen[3], Yihua An[4*]

[1]Dougezhuang Community Health Service Center, Chaoyang District, Beijing 100023, China.

[2]Key Laboratory of Zoological Systematics and Evolution, Institute of Zoology, Chinese Academy of Sciences, Beijing 100101, China.

[3]The Fifth Medical Center of the General Hospital of the People's Liberation Army, 8 East St, Fengtai District, Beijing 100071, China.

[4]Department of Neurosurgery, Sanbo Brain Hospital, Capital Medical University, Beijing 100093, China.

*** Correspondence:**
Yihua An
riveran@ccmu.edu.cn

Supplementary Figures and Tables

1. Supplementary Figures

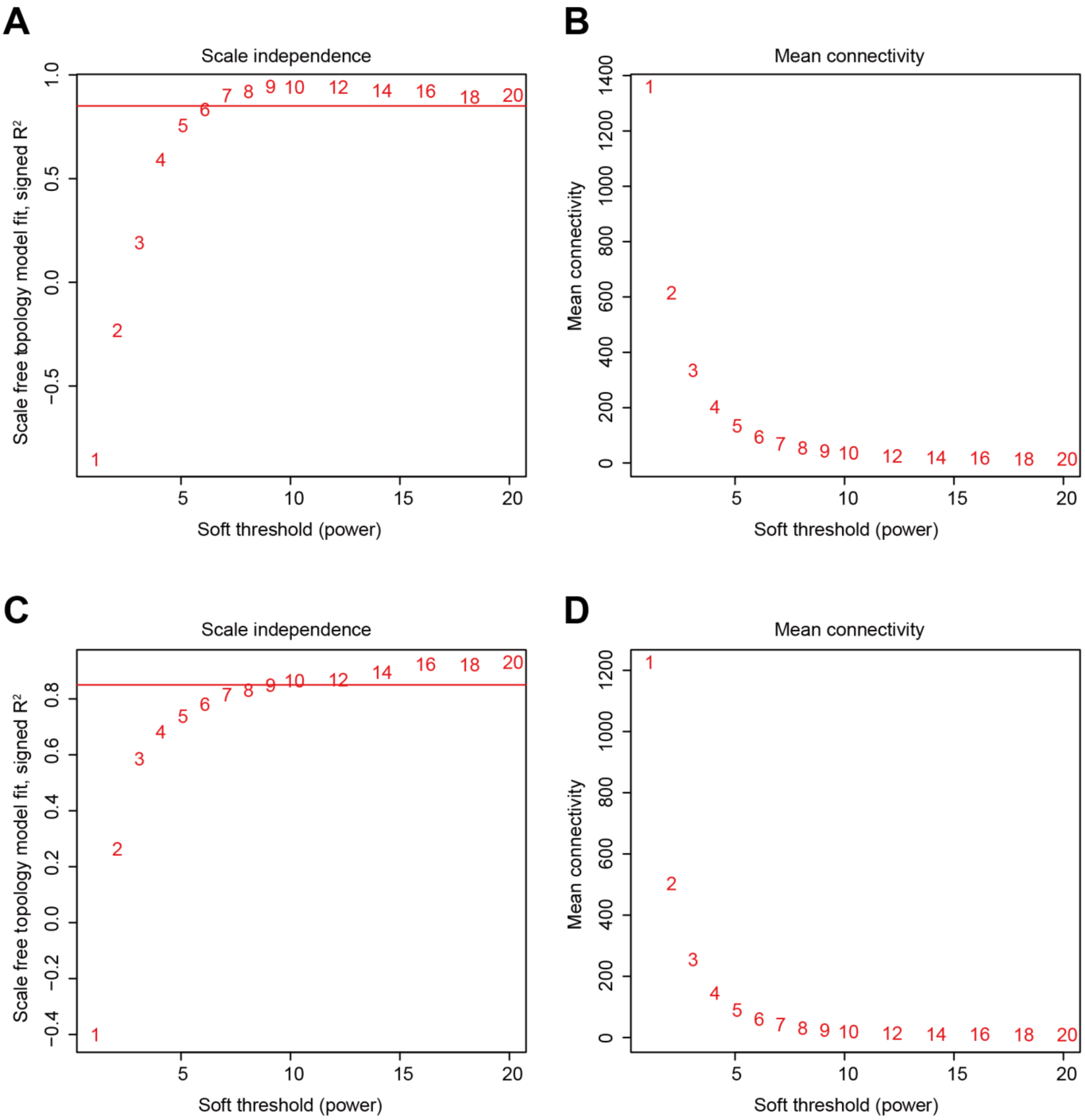

Supplementary Figure 1. Selection of the soft thresholds for AIS and AMI. (A) Analysis of the scale-free index for AIS. **(B)** Analysis of the mean connectivity for AIS. **(C)** Analysis of the scale-free index for AMI. **(D)** Analysis of the mean connectivity for AMI.

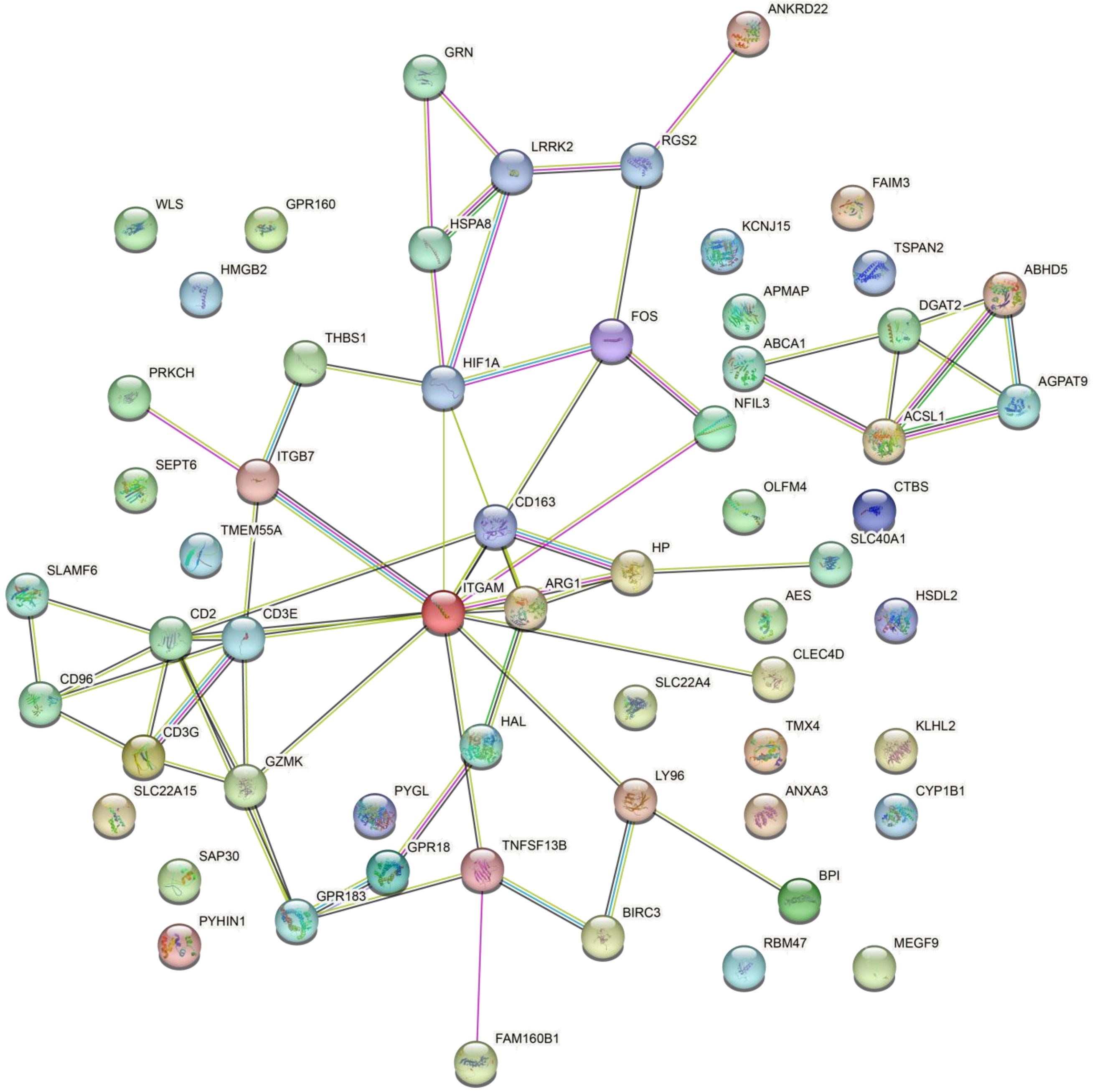

Supplementary Figure 2. The PPI network of the 60 key genes in STRING.

2. Supplementary Tables

Supplementary Table 1. The GO terms shared by all four datasets.

Ontology	ID	Description	Count (DEGs of AIS)	Count (hub genes of AIS)	Count (DEGs of AMI)	Count (hub genes of AMI)
BP	GO:0001819	positive regulation of cytokine production	43	122	41	156
BP	GO:1903131	mononuclear cell differentiation	41	122	40	153
BP	GO:0030098	lymphocyte differentiation	38	108	37	145
BP	GO:0050867	positive regulation of cell activation	37	107	36	122
BP	GO:0002696	positive regulation of leukocyte activation	37	105	34	118
BP	GO:0045785	positive regulation of cell adhesion	35	108	26	133
BP	GO:0022407	regulation of cell-cell adhesion	35	113	25	132
BP	GO:0032103	positive regulation of response to external stimulus	34	111	29	136
BP	GO:0050863	regulation of T cell activation	32	97	27	115
BP	GO:0007159	leukocyte cell-cell adhesion	32	101	29	133
BP	GO:0051251	positive regulation of lymphocyte activation	31	92	27	101
BP	GO:0002768	immune response-regulating cell surface receptor signaling pathway	31	81	22	107
BP	GO:0002443	leukocyte mediated immunity	31	119	30	115
BP	GO:0002253	activation of immune response	31	88	22	118
BP	GO:1903037	regulation of leukocyte cell-cell adhesion	30	93	23	113
BP	GO:0002460	adaptive immune response based on somatic recombination of immune receptors built from immunoglobulin superfamily domains	29	87	22	89
BP	GO:0030217	T cell differentiation	28	76	26	113
BP	GO:0022409	positive regulation of cell-cell adhesion	28	81	20	102
BP	GO:0002831	regulation of response to biotic stimulus	28	100	25	106
BP	GO:0002697	regulation of immune effector process	27	101	30	113
BP	GO:1903039	positive regulation of leukocyte cell-cell adhesion	26	73	19	93
BP	GO:0070661	leukocyte proliferation	26	84	34	103
BP	GO:0031349	positive regulation of defense response	26	81	22	96

BP	GO:0002757	immune response-activating signal transduction	26	71	18	96
BP	GO:0002449	lymphocyte mediated immunity	26	91	22	80
BP	GO:0002429	immune response-activating cell surface receptor signaling pathway	26	71	18	96
BP	GO:1903706	regulation of hemopoiesis	25	88	31	116
BP	GO:0050870	positive regulation of T cell activation	25	70	18	83
BP	GO:0046651	lymphocyte proliferation	25	78	33	92
BP	GO:0032943	mononuclear cell proliferation	25	78	33	94
BP	GO:0030099	myeloid cell differentiation	25	84	27	103
BP	GO:0002683	negative regulation of immune system process	25	100	31	112
BP	GO:0002237	response to molecule of bacterial origin	25	84	27	88
BP	GO:0032496	response to lipopolysaccharide	24	79	27	82
BP	GO:0002366	leukocyte activation involved in immune response	23	87	30	101
BP	GO:0002263	cell activation involved in immune response	23	87	30	101
BP	GO:0045088	regulation of innate immune response	22	67	21	78
BP	GO:0002274	myeloid leukocyte activation	20	65	27	84
BP	GO:1902105	regulation of leukocyte differentiation	19	72	23	94
BP	GO:0070663	regulation of leukocyte proliferation	19	66	28	77
BP	GO:0050670	regulation of lymphocyte proliferation	19	61	28	71
BP	GO:0048872	homeostasis of number of cells	19	69	19	90
BP	GO:0032944	regulation of mononuclear cell proliferation	19	61	28	72
BP	GO:0002703	regulation of leukocyte mediated immunity	19	72	19	83
BP	GO:0002221	pattern recognition receptor signaling pathway	19	43	14	66
BP	GO:0002833	positive regulation of response to biotic stimulus	18	57	17	64
BP	GO:0001906	cell killing	18	65	18	56
BP	GO:0070665	positive regulation of leukocyte proliferation	17	42	16	47
BP	GO:0050671	positive regulation of lymphocyte proliferation	17	40	16	42
BP	GO:0046631	alpha-beta T cell activation	17	52	14	64
BP	GO:0034612	response to tumor necrosis factor	17	59	15	56
BP	GO:0032946	positive regulation of mononuclear cell proliferation	17	40	16	43
BP	GO:0002699	positive regulation of immune effector process	17	74	16	74
BP	GO:0002573	myeloid leukocyte differentiation	17	51	17	58
BP	GO:0002285	lymphocyte activation involved in immune response	17	60	20	69
BP	GO:0042098	T cell proliferation	16	60	18	68

BP	GO:0002706	regulation of lymphocyte mediated immunity	15	55	12	55
BP	GO:0001909	leukocyte mediated cytotoxicity	14	49	15	45
BP	GO:0042129	regulation of T cell proliferation	13	48	16	53
BP	GO:0002224	toll-like receptor signaling pathway	13	33	10	42
BP	GO:2000377	regulation of reactive oxygen species metabolic process	12	36	11	37
BP	GO:0071887	leukocyte apoptotic process	12	38	10	43
BP	GO:0045089	positive regulation of innate immune response	12	42	13	50
BP	GO:0032649	regulation of interferon-gamma production	12	33	15	42
BP	GO:0032609	interferon-gamma production	12	33	15	42
BP	GO:0030183	B cell differentiation	12	39	13	51
BP	GO:0070227	lymphocyte apoptotic process	11	27	8	31
BP	GO:0002367	cytokine production involved in immune response	11	36	10	36
BP	GO:0002286	T cell activation involved in immune response	11	32	14	44
BP	GO:0035710	CD4-positive, alpha-beta T cell activation	10	31	10	42
BP	GO:0031341	regulation of cell killing	10	36	12	38
BP	GO:0070228	regulation of lymphocyte apoptotic process	9	20	7	29
BP	GO:0032729	positive regulation of interferon-gamma production	9	25	11	28
BP	GO:0001910	regulation of leukocyte mediated cytotoxicity	9	31	11	31
BP	GO:0050854	regulation of antigen receptor-mediated signaling pathway	8	22	8	28
BP	GO:0032655	regulation of interleukin-12 production	8	23	8	26
BP	GO:0032615	interleukin-12 production	8	23	8	26
CC	GO:0060205	cytoplasmic vesicle lumen	31	101	26	100
CC	GO:0031983	vesicle lumen	31	101	26	100
CC	GO:0034774	secretory granule lumen	30	99	26	100
CC	GO:0030667	secretory granule membrane	30	74	33	129
CC	GO:0030055	cell-substrate junction	28	101	31	106
CC	GO:0005925	focal adhesion	28	101	31	103
CC	GO:0030139	endocytic vesicle	26	79	19	99
CC	GO:0098857	membrane microdomain	24	66	19	91
CC	GO:0070820	tertiary granule	24	57	25	90
CC	GO:0098852	lytic vacuole membrane	23	108	21	115
CC	GO:0045121	membrane raft	23	65	19	90
CC	GO:0005774	vacuolar membrane	23	116	25	130

CC	GO:0005765	lysosomal membrane	23	108	21	115
CC	GO:0101002	ficolin-1-rich granule	21	59	25	81
CC	GO:0042581	specific granule	21	61	26	83
CC	GO:0030666	endocytic vesicle membrane	17	47	14	48
CC	GO:1904813	ficolin-1-rich granule lumen	11	40	14	44
CC	GO:0070821	tertiary granule membrane	11	23	12	41
CC	GO:0035579	specific granule membrane	11	30	14	46
CC	GO:0101003	ficolin-1-rich granule membrane	10	19	11	37
CC	GO:1904724	tertiary granule lumen	9	21	11	29
CC	GO:0035580	specific granule lumen	9	29	11	32
CC	GO:0001772	immunological synapse	7	21	6	22

Terms in yellow are related to immunity.
